# Conventional specimen radiography in breast-conserving therapy: a useful tool for intraoperative margin assessment after neoadjuvant therapy?

**DOI:** 10.1007/s10549-023-06976-2

**Published:** 2023-06-11

**Authors:** Benedikt Schäfgen, Annabelle Haller, Hans-Peter Sinn, Manuel Feisst, Christina Gomez, Anne Stieber, Juliane Nees, Riku Togawa, André Pfob, André Hennigs, Johanna Hederer, Fabian Riedel, Sarah Fastner, Jörg Heil, Michael Golatta

**Affiliations:** 1 Department of Gynecology and Obstetrics, Breast Unit, University Hospital, 69120 Heidelberg, Germany; 2 Department of Pathology, University of Heidelberg, Heidelberg, Germany; 3 Institute for Medical Biometry, University of Heidelberg, INF 130.3, 69120 Heidelberg, Germany; 4 Brustzentrum Heidelberg Klinik St. Elisabeth, Max-Reger-Straße 5-7, 69121 Heidelberg, Germany; 5 Department of Radiology, University of Heidelberg, Heidelberg, Germany; 6 Institute of Pathology, University Hospital, INF 224, 69120 Heidelberg, Germany; 7 Department of Diagnostic and Interventional Radiology, University Hospital, INF 110, 69120 Heidelberg, Germany

**Keywords:** Breast cancer, Breast conserving therapy, Surgical margins, Intraoperative re-excision, Specimen radiography, Neoadjuvant chemotherapy

## Abstract

**Purpose:**

A previous study in our breast unit showed that the diagnostic accuracy of intraoperative specimen radiography and its potential to reduce second surgeries in a cohort of patients treated with neoadjuvant chemotherapy were low, which questions the routine use of Conventional specimen radiography (CSR) in this patient group. This is a follow-up study in a larger cohort to further evaluate these findings.

**Methods:**

This retrospective study included 376 cases receiving breast-conserving surgery (BCS) after neoadjuvant chemotherapy (NACT) of primary breast cancer. CSR was performed to assess potential margin infiltration and recommend an intraoperative re-excision of any radiologically positive margin. The histological workup of the specimen served as gold standard for the evaluation of the accuracy of CSR and the potential reduction of second surgeries by CSR-guided re-excisions.

**Results:**

362 patients with 2172 margins were assessed. The prevalence of positive margins was 102/2172 (4.7%). CSR had a sensitivity of 37.3%, a specificity of 85.6%, a positive predictive value (PPV) of 11.3%, and a negative predictive value (NPV) of 96.5%. The rate of secondary procedures was reduced from 75 to 37 with a number needed to treat (NNT) of CSR-guided intraoperative re-excisions of 10.

In the subgroup of patients with clinical complete response (cCR), the prevalence of positive margins was 38/1002 (3.8%), PPV was 6.5% and the NNT was 34.

**Conclusion:**

This study confirms our previous finding that the rate of secondary surgeries cannot be significantly reduced by CSR-guided intraoperative re-excisions in cases with cCR after NACT. The routine use CSR after NACT is questionable, and alternative tools of intraoperative margin assessment should be evaluated.

## Introduction

Nowadays, most patients with high-risk early breast cancer are treated with neoadjuvant chemotherapy (NACT) followed by breast-conserving surgery (BCS) and adjuvant radiotherapy. This approach leads to a significant reduction of surgical invasiveness, and hence, treatment morbidity, with equal [1, 2] or superior [3, 4] overall survival compared to mastectomy. The combination of NACT and BCS leads to an improvement of the esthetic outcome and patient satisfaction [5–7] as well as a higher quality of life [8, 9] compared to mastectomy. For an optimal esthetic outcome, the surgeon should avoid removing too much healthy tissue, but the resection margins of the tumor bed should be tumor free to limit the risk of local recurrence [10]. In the clinical routine, the specimen is evaluated radiologically with mammography in two orientations (conventional specimen radiography, CSR), and the radiologist recommends the surgeon to perform an intraoperative re-excision if the margins seem to be tumor infiltrated. Ideally, this leads to tumor-free resection margins and can help to avoid a secondary re-excision. Cicarelli et al. report a reduction of the reintervention rate from 31 to 20% through CSR-guided re-excisions in a cohort of 123 patients undergoing breast-conserving surgery [11]. Naz et al. also describe that CSR was as a “sufficient tool” to predict the margin status with a positive predictive value of 83.3%, a sensitivity of 80.7%, and a specificity of 81% [12]. Yet the proportion of patients who actually benefit from CSR depends on the prevalence of initially positive margins. Thanks to more frequent administration and more effective systemic treatment options, an increasing number of patients achieve a pathological complete response (pCR) after NACT [13]. Thus, the prevalence of initially positive margins is expected to be lower in patients after NACT and the use of CSR seems questionable in the postneoadjuvant setting [14]. In this study, we evaluated the efficacy of CSR for the diagnosis of infiltrated resection margins and the potential reduction of the reintervention rate through CSR-guided intraoperative re-excisions.

## Material and methods

This study was approved by the ethics committee of the University’s Medical Faculty under file number S-729/2020. Obtaining patient consent was waived for anonymized, retrospective analysis by the ethics committee.

### Patient population

**Fig. 1:**
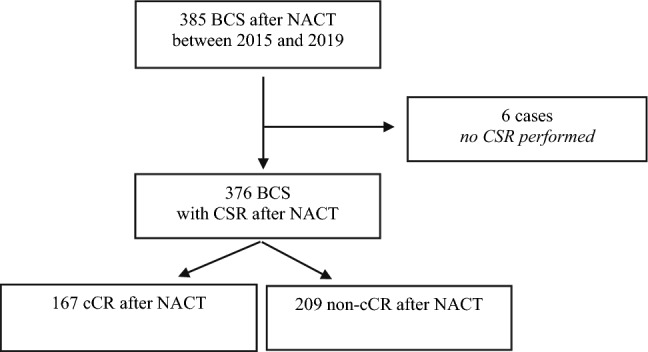
Flow diagram of patient population. 385 patients planned for breast conserving surgery (BCS) with conventional specimen radiography (CSR) after neoadjuvant chemotherapy (NACT) were included in the retrospective analysis. For further analysis, they were divided in two subgroups with clinical complete response (cCR) and non-cCR

Patients treated at the Breast Unit with BCS after NACT of invasive breast cancer between July 2015 and June 2019 were included consecutively in the retrospective analysis. Cases that did not receive CSR (*n* = 6), mostly for reasons of good tumor palpability were excluded from further analysis. For subgroup analysis, patients´ response to NACT was classified as clinical complete response (cCR; defined as the absence of evidence of residual tumor in clinical examination, ultrasound, and mammography after NACT) and non-cCR (Fig. 1).

### Conventional specimen radiography and surgical procedure

Preoperative wire localization using ultrasound or stereotactic guidance was performed and controlled by mammography.

The specimen orientation was marked by sutures of different lengths according to institutional standard operating procedures. CSR was performed in the breast unit using Mammomat Inspiration (Siemens AG, Erlangen, Germany) with 1.4 × direct magnification in two orthogonal views without compression. One of six physicians with more than 10 years of experience in diagnostic mammography and CSR evaluated the position of the target lesion and its relation to the resection margins. Clinical history and previous images were available to the radiologist. If any of the margins appeared to be infiltrated, the radiologist advised the surgeon to perform an intraoperative re-excision of the same orientation.

**Fig. 2:**
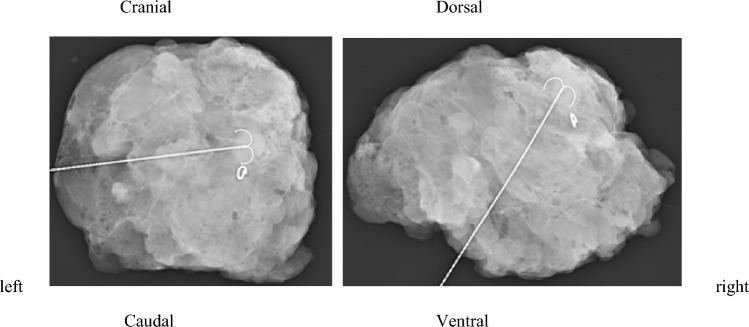
Example of a conventional two-view specimen radiography (CSR) of a patient with residual tumor (non-clinical complete response, non-cCR), performed using a Mammomat Inspiration device (Siemens AG, Erlangen, Germany) with 1.4 × direct magnification. Marking wire and clipmarker are visible in the former tumor bed. The tumor bed seems to reach the dorsal resection margin, so re-excision was recommended based on CSR. In contrast, the pathological workup showed a pathologic complete response (pCR), indicating a false-positive CSR

The pathologic workup of the specimen and the re-excisions was the gold standard for the evaluation of the diagnostic accuracy of CSR. For patients treated between 2014 and 2017, a negative margin was defined as > 1 mm in invasive carcinoma and > 2 mm in ductal carcinoma in situ (DCIS) according to the national guideline of 2012 [15]. For patients treated after December of 2017, a clear margin was defined as “no ink on tumor,” according to the new national guideline [16] (Fig. 2).

### Statistical analysis

Descriptive analyses were performed using IBM SPSS Statistics Version 26, (Armong, NY, USA). Sensitivity and specificity of CSR were calculated along with 95% confidence intervals using SAS 9.4 WIN (Cary, NC, USA).

The primary endpoint was the number needed to treat (NNT), defined as the inverse of the absolute risk reduction to avoid a second surgery by CSR-guided intraoperative resections. Subgroup analysis was performed for cCR and non-cCR patients separately.

## Results

362 patients received BCS after NACT and were included in this analysis (Table 1). For each primary resection specimen, six margins were assessed (2172 in total). Most patients had invasive-ductal carcinoma (NST; *n* = 348, 96.1%), and the most prevalent grading was G3 (*n* = 218, 60.2%). (Table 1).

**Table 1:** Patient and tumor characteristics of 362 breast cancer patients recruited between 2015 and 2019 for the analysis of the efficacy of cSR-guided re-excisions in breast-conserving surgery after neoadjuvant chemotherapy

Number of patients	(*n* = 362) (Percentages in brackets)
Age	
Median	54
Interquartile	46; 61
Range of 25 and 75 percentile race/ethnicity	Not systematically assessed, mostly European
Menopausal status	
Premenopausal Perimenopausal Postmenopausal	117 (32.3) 55 (15.2) 190 (52.5)
Breast density	
(ACR)	
A	32 (8.8)
B	161 (44.5)
C	108 (29.8)
D	19 (5.2)
Histological tumor type	
Invasive-ductal (NST)	348 (96.1)
Invasive-lobular	8 (2.2)
Invasive-medullary	1 (0.3)
Invasive-mucinous	1 (0.3)
Missing data	4 (1.1)
Grading	
G1	4 (1.1)
G2	137 (37.8)
G3	218 (60.2)
Missing data	3 (0.8)
Target structure for lesion marking Clip marker	354 (97.8)
Microcalcifications	100 (27.6)
Radiographic presentation of the tumor	
Only mass	253 (69.9)
Mass with microcalcifications	100 (27.6)
Only microcalcifications	4 (1.1)
Undefined	5 (1.3)
MRI performed	
Before and after NACT	25 (6.9)
Only before NACT	75 (26.2)
Only after NACT	35 (9.7)
Not performed	227 (62.7)
Remission status	
cCR	167 (46.1)
With pCR (% of cCR patients)	102 (61.1)
Non-cCR	195 (53.9)
With pCR (% of non-cCR patients)	48 (24.6)
Final T-category (ypT)	
0 is 1 −1mic −1a −1b −1c 2 3 4	151 (41.7) 19 (5.2) 5 (1.4) 47 (13.0) 43 (11.8) 61 (16.9) 34 (9.4) 2 (0.6) 0 0
Median specimen weight (grams) Primary resection re-Resection	30.0 (interquartile range: 20;49) 6.0 (interquartile range: 3; 14)
Histologically Infiltrated margins by orientation	
Total	102 (4.7)
Medial	21 (20.6)
Lateral	17 (16.7)
Kranial	29 (28.4)
Kaudal	10 (9.8)
Ventral	11 (10.8)
Dorsal	14 (13.7)

*cCR* clinical complete response, *pCR* pathological complete response

167 Patients (46.1%) had a clinical complete response (cCR), 195 (53.9%) had no clinical complete response (non-cCR). In 150 cases (41.4%), NACT resulted in a pCR (ypT0). 102 of the cCR patients (61.1%) and 48 of the non-cCR patients (24.6%) had a pCR.

### Histopathological margin infiltration by orientations

The histopathological workup of the main specimen (without re-excisions) showed an infiltration of 102 (4.7%) margins. The medial and cranial orientation most frequently showed margin involvement (50 positive margins, 2.3%).

### Margin assessment by CSR

In total, 2172 margins were analyzed with CSR (Table 2), of which 336 (15.5%) were radiologically positive. 38 (1.7%) were histologically and radiologically positive (true positive CSR). 298 (13.7%) histopathologically clear margins were falsely assessed as positive by CSR. In these cases, healthy tissue was re-resected unnecessarily if the surgeon followed the recommendation for re-excision. Of 1836 radiologically negative margins, 64 (2.9%) were histologically infiltrated (false negative CSR). In these cases, no recommendation for re-excision was given based on CSR, and residual tumor was missed in the first surgery (unless the surgeon performed a re-excision without a CSR recommendation).

**Table 2:** Evaluation of Conventional Specimen Radiography on a margin level for the whole and for patients with clinical complete response versus no clinical complete response

	Overall cohort		Clinical complete response (cCR)	
	Pathologic assessment positive*	Pathologic assessment negative**	Pathologic assessment positive*	Pathologic assessment negative**
CSR positive	38	298	9	130
CSR negative	64	1772	29	834
Total––no. (%)	2172 (100%)		1002 (100%)	

		(95% CI)		(95% CI)
Sensitivity––% (95% CI)	37.3%	(35.3–39.3%)	23.7%	(21.1–26.3%)
Specificity––% (95% CI)	85.6%	(84.1–87.1%)	86.5%	(84.4–88.6%)
PPV––% (95% CI)	11.3%	(9.8–12.77)	6.5%	(5.0–8.0%)
NPV––% (95% CI)	96.5%	(95.0–98.0%)	96.6%	(95.5–97.7%)
Margin conversion through CSR––no. (%)	21	(1.0%)	5	(0.5%)
NNT		101		200

*cCR* clinical complete response, *CSR* Conventional Specimen Radiography, *NACT* neoadjuvant chemotherapy, *NNT* number needed to treat, *NPV* negative predictive value, *PMR* positive margin rate, *PPV* positive predictive value

*Tumor-infiltrated margin in histopathologic evaluation of the surgical specimen

**No tumor-infiltrated margin in histopathologic evaluation of the surgical specimen

### Comparison of margin assessment between the whole cohort and the subgroup of cCR patients

Regarding all 2172 margins, CSR had a sensitivity of 37.3%, specificity of 85.6%, PPV of 11.3%, and NPV of 96.5%. In the subgroup of cCR patients, the prevalence of histologically positive margins was 38 of 1002 (3.8%). 9 of these margins were correctly diagnosed as radiologically positive (prevalence of true positive margins in CSR, 0.9%). In contrast, 130 of 964 histologically negative margins were positive in CSR (prevalence of false-positive margins in CSR: 13.0%).

### Intraoperative re-excisions and final-positive margin status on case level

In 212 (58.6%) patients, at least one intraoperative re-excision was performed *(*Table 3*)*. In 177 (83.5%) cases, all margins were histopathologically negative, indicating that the re-excision was unnecessary. In 35 (9.7%) cases, infiltration of at least one margin was confirmed in histopathological examination. In 19 (5.2%) cases, all histologically infiltrated margins were correctly identified by CSR. In the remaining 16 (4.4%) cases, at least one histologically infiltrated margin was missed by CSR. Through intraoperative re-excisions, the number of infiltrated margins could be reduced from initially 35 (9.7%) to 16 (4.4%).

**Table 3:** Effect of CSR-guided re-resections on the final margin status and reduction of secondary surgeries on case level (*n* = 362)

	Overall cohort	cCR after NACT	Non-cCR after NACT
Number of cases	362 (100%)	167 (100%)	195 (53.9%)
Initial PMR (CSR)	194 (53.6%)	82 (49.1%)	112 (57.4%)
True positive	35 (9.7%)	10 (6.0%)	25 (12.8%)
False positive	159 (43.9%)	72 (43.1%)	87 (44.6%)
true negative	150 (41.4%)	75 (44.9%)	75 (38.5%)
False negative	18 (5.0%)	10 (6.0%)	8 (4.1%)
Sensitivity	66.0%	50.0%	75.8%
Specificity	48.5%	51.0%	46.3%
PPV	18.0%	12.2%	22.3%
NPV	89.3%	88.2%	90.4%
Final PMR (pathologic assessment)	16 (4.4%)	6 (3.6%)	10 (5.1%)
Conversion of margin status through CSR	19 (5.2%)	5 (3.0%)	14 (7.2%)
NNT for conversion of margin status through CSR	20	34	14
secondary surgeries	37 (10.2%)	8 (4.8%)	29 (14.9%)
Number of secondary surgeries avoided through CSR (absolute risk reduction)	38 (10.5%)	5(3.0%)	33 (16.9%)
NNT to avoid secondary surgeries through CSR	10	34	6

Patients with a cCR have a lower prevalence of initially positive resection margins and a higher number needed to treat to avoid secondary surgeries through CSR-guided intraoperative re-excisions

*cCR* clinical complete response, *CSR* Conventional Specimen Radiography, *NACT* neoadjuvant chemotherapy, *NNT* number needed to treat, *PMR* positive margin rate in histology

### Effect of CSR-guided resections on secondary procedures

Through intraoperative re-excisions based on CSR together with the gross assessment by the surgeon, only 37 patients required a second surgery. Without intraoperative re-excisions, 75 patients would have required further surgery. Thus, 38 secondary surgeries were avoided through intraoperative re-excisions, and the rate of secondary procedures was reduced by 49.3%, resulting in a NNT of 10. In the cCR subgroup, the number of secondary surgeries was reduced from 13 to 8 patients by CSR-guided re-excisions. This translates to a NNT (avoid one second surgery) of 34 in the cCR subgroup.

Table 3 shows the effect of CSR-guided re-resections on the final margin status and reduction of secondary surgeries on case level.

### Detection of clip markers by CSR

344 of 354 (97.2%) clip markers used for preoperative localization were detected in CSR. In 10 cases, the clip marker was detected neither in CSR nor in a postsurgical control mammography (therefore, the clip must have been removed during the surgery without noticing by the surgeon).

## Discussion

Despite its widespread use in the clinical routine, there is still limited evidence for the efficacy of CSR. Many studies are limited to the analysis of either the margin level or the case level. In our previous study which integrated both margin and case level, we found that the low prevalence of initially infiltrated margins in combination with the mediocre sensitivity of CSR resulted in a small potential to reduce the rate of second surgeries. From these results, we concluded that the routine use of CSR-guided re-excisions lacks evidence and designed this follow-up study.

### Comparison of this analysis with a previous study on the use of CSR after NACT

As this study was designed as a follow-up study of the previous cohort of patients treated with NACT, we compared the results of both studies.

The sample size in the current study was 2.1 times larger than the previous analysis. We found a higher sensitivity (66.0% vs. 52.0%) and PPV (18.0% vs. 17.3%) in the overall cohort and in the cCR subgroup (50.0% vs. 42.9%; 12.2% vs. 8.3%, respectively). Accordingly, the NNT to avoid a second surgery by CSR-guided intraoperative re-excisions was lower in the present study (10 vs. 25 in all patients after NACT, and 34 vs. 91 in the cCR subgroup). (Table 4).

**Table 4:** Comparison of the results on case level of the present study with 362 patients treated with NACT and BCS between 2015 and 2019 to the analysis 174 patients treated between 2014 and 2015 (Data source: Schaefgen et al., Does conventional specimen radiography after neoadjuvant chemotherapy of breast cancer help to reduce the rate of second surgeries? Breast Cancer Res Treat. 2022 Feb; PMID: 34878635)

Study cohort	2022 (Present study)		2021 (Schaefgen et al BCRT 2022)	
*n* =	362		174	
sensitivity		66.0%		52.0%
PPV		18.0%		17.3%
secondary surgeries	37	10.2%	16	9.2%
number of secondary surgeries avoided through CSR	75	20.7%	23	13.2%
NNT to avoid secondary surgeries through CSR	10		25	

cCR: clinical complete response, CSR: Conventional Specimen Radiography, NACT: neoadjuvant chemotherapy, NNT: number needed to treat, PPV: positive predictive value

In summary, we found a higher diagnostic accuracy of CSR and consequently a lower NNT of 10 (vs. 25 in the pre-study) to avoid one second surgery of 10 for the overall cohort in the present study. This means that 9 of 10 patients would not benefit from CSR, while one second surgery could be avoided. From the clinical perspective, this seems to be acceptable, because the potential harm of an unnecessary CSR is limited to unnecessary removal of a small amount of healthy tissue if a re-excision is recommended based on false-positive CSR. [17].

However, in the subgroup of cCR patients, the number needed to treat was 34, meaning that 33 patients did not benefit from CSR (and potentially healthy tissue was resected unnecessarily), while only one secondary surgery was avoided. It seems reasonable that if the preoperative imaging (including sonography and mammography) shows no evidence of residual tumor (cCR), CSR will also be negative. In such a case, the patient has a high chance of a pCR, and even if there is residual tumor (non pCR), the disease is obviously mammographically occult. The NNT in the cCR subgroup found in the current study was substantially lower than in the pre-study (34 vs. 91), but in our opinion, the NNT is still too high to justify the routine use of CSR-guided re-excision in all patients with a cCR. In fact, when costs and risks of CSR-guided re-resections are balanced, CSR does not seem to be an appropriate tool for margin assessment in cCR patients.

From this perspective, it would be a pragmatic approach to perform CSR in cCR patients only to prove the removal of the clip marker, but not routinely for margin assessment and the recommendation of targeted re-excisions. As the results of this study showed, CSR was a reliable tool for the detection of the clip markers with a detection rate of 97.2%, but unable to reduce the rate of second surgeries. In selected cCR cases, CSR-guided re-excisions may still be useful to indicate targeted re-excisions. For example, in cases with a large regressive tumor bed or difficult margin preparation due to the localization of the lesion (e.g., close to the skin or thoracic wall), the surgeon should have the possibility to ask the radiologist for a detailed margin assessment of the CSR and potential recommendations for intraoperative re-excision.

#### Comparison of CSR to other approaches to reduce the rate of infiltrated resection margins

Other approaches to reduce the rate of positive resection margins include alternative methods of intraoperative identification of infiltrated margins (by imaging, histological analysis, or MarginProbe) and intraoperative re-excisions without intraoperative margin assessment (e.g., complete cavity-shaved margins).

Perera et al. analyzed the accuracy of breast specimen ultrasound (SUS) in 99 lesions (384 margins) and found a PPV for predicting infiltrated margins of 16% (95% CI 5–34), and NPV of 99% (95% CI 97–99), which is similar to the PPV and NPV of CSR found in the present study (11.3 and 96.5%, respectively)[18]. In a large meta-analysis on intraoperative margin assessment techniques, St. John et al. also report a similar accuracy of intraoperative ultrasound with a sensitivity of 59% and a specificity of 81%, (pooled from four studies) compared to specimen radiography (sensitivity of 53%, specificity of 84%, pooled from nine studies). In this analysis, imaging techniques were inferior to intraoperative histological analysis of frozen section (sensitivity of 86%, specificity of 96%, pooled from nine studies) and cytology (sensitivity of 91% and specificity of 95%, pooled from eleven studies). On the other hand, procedures including histological/cytological margin assessment are more time consuming and cost intensive [19]. The ability of margin probe (a procedure involving radiofrequency spectroscopy of the margin surface) is also a matter of ongoing debate. LeeVan et al. report a mediocre sensitivity for the intraoperative detection of positive margins using MarginProbe of 67% and specificity of 60%, a PPV of 16%, and a NPV of 94%, which is similar to the results we found for CSR, while Schnabel et al. report higher accuracy and ability to reduce second surgeries through MarginProbe [20, 21].

Routinely cavity-shaved margins (CS) can be an alternative approach to targeted re-excisions based on intraoperative margin assessment. Chagpar et al. report a significantly lower rate of positive margins in the group with CS than in the no-shave group (19% vs. 34%, *P* = 0.01), as well as a lower rate of second surgery for margin clearance (10% vs. 21%, *P* = 0.02) [22]. Similarly, in a meta-analysis on the effect of routinely shaved margins, Fernandez-Pacheco et. al. report a tendency to lower re‐excision rates and lower histologic margin positivity in 17 of 22 studies. Yet the authors point out that the majority of studies did not find a statistically significant reduction of re‐excision rates for CS [23].

In summary, the accuracy of CSR is comparable to other tools of margin assessment. There is no consensus on the ideal technique of intraoperative resections, and further studies are needed to improve the level of evidence in this field.

### False positive rate (FPR)

In this analysis, the initially positive margin rate on case level was 14.6% (53 primary resectates had at least one infiltrated margin). Of 2172 margins, 102 (4.7%) were initially positive. The false-positive rate on margin level was 13.8% (298 of 2172), which is in line with the previous analysis in the smaller cohort with an FPR of 10.3%. We assume that the most important factor contributing to a high false-positive rate is that NACT induces regressive changes (necrosis and fibrosis) in the tumor bed, which can appear like residual tumor [24, 25].

A false-positive CSR can lead to the unnecessary removal of healthy tissue, if the surgeon follows the recommendation to perform a re-excision, which can result in a poorer esthetic outcome. One potential explanation for the relatively high false-positive rate is the so-called pancake phenomenon, as described by Graham et al.: Upon excision, the tensions in the tumor-bed tissue lead to shrinkage of the resectate, and the radiological margin width appears smaller than it was in situ, and margins can falsely appear to be tumor-infiltrated. This effect is amplified, if inadequate pressure is applied while the CSR radiograms are taken [26]. An important source of error for false-positive and false-negative radiological margin assessment is the orientation of the specimen. In our institution, the specimen orientation is routinely marked using threads of different lengths in situ before the tissue is excised or directly after removal of the specimen to allow clear radiological and histological margin identification. Yet, especially small resectates can accidentally be twisted upon removal or the three-dimensional shape of the specimen can be misinterpreted in the two-dimensional radiogram [27].

### Limitations

To the best of our knowledge, this is the largest analysis on CSR efficacy in a NACT-treated cohort. Yet, due to the retrospective design of the study, the conclusions have to be confirmed in a prospective trial before recommending a change of practice.

For a comprehensive assessment of CSR accuracy, we decided to analyze the results both on margin level and on case level. However, regarding the results on margin level, one has to be aware that the six margins of each specimen cannot be regarded as independent variables, because they share patient-specific properties (e.g., density of the breast tissue).

One limitation in the study design is that re-excisions were performed according to the surgeon's discretion based on his/her clinical impression and the result of CSR. There was no systematic assessment on how strongly his/her decision was influenced by the CSR-based recommendation. It is possible that in some of the cases, the surgeon would have performed an intraoperative re-excision independently from the CSR recommendation, based on his/her subjective assessment.

## Conclusion

In this follow-up study in a larger cohort of patients after NACT, the diagnostic performance of CSR was slightly higher than in the previous analysis. Yet the NNT to avoid one second surgery is still high, especially after cCR, due to the low prevalence of initially positive margins. To avoid overtreatment in form of unnecessary tissue removal, the use of CSR-guided re-excisions cannot be generally recommended after NACT and should be limited to cases with residual tumor in preoperative imaging (non-cCR) or used only to confirm that a clip marker was removed completely.

## Data Availability

Upon request from the corresponding author.

## Abbreviations

ACR: American College of Radiology
BCS: Breast conserving surgery
cCR: Clinical complete response
CI: Confidence interval
CSR: Conventional Specimen Radiography
NACT: Neoadjuvant chemotherapy
NNT: Number needed to treat
NPV: Negative predictive value
pCR: Pathological complete response
PMR: Positive margin rate
PPV: Positive predictive value
